# Assessment of Neurological Status in Patients with Cerebrovascular Diseases through the Nursing Outcome Classification: A Methodological Study

**DOI:** 10.3390/nursrep12010016

**Published:** 2022-03-02

**Authors:** Danielle Uehara de Lima, Rafaella Pessoa Moreira, Tahissa Frota Cavalcante, Renata Cristina Gasparino, Suellen Cristina Dias Emidio, Ana Railka de Souza Oliveira-Kumakura

**Affiliations:** 1 Nursing Department, Clinical Hospital of University of Campinas, Campinas 13083-887, SP, Brazil; danielleuehara@hc.unicamp.br; 2 Health Sciences Institute, University for International Integration of the Afro-Brazilian Lusophony (UNILAB), Redencao 62790-000, CE, Brazil; rafaellapessoa@unilab.edu.br (R.P.M.); tahissa@unilab.edu.br (T.F.C.); 3 School of Nursing, University of Campinas, Campinas 13083-887, SP, Brazil; grenata@unicamp.br; 4 Nursing Department, University of Tocantins, Palmas 77650-000, TO, Brazil; suellen.emidio@ufjf.edu.br

**Keywords:** validation studies, scales, patient outcomes, nursing-sensitive outcomes, neurologic examination, cerebrovascular diseases

## Abstract

Nurses play an important role in healthcare, and the Nursing Outcomes Classification is a key tool for the standardization of care. This study aims to validate the nursing outcome “Neurological Status” for patients with cerebrovascular diseases. A methodological study was performed in four phases. In Phase 1, the relevance of the indicators was evaluated by seven specialists and the modified kappa coefficient and content validity index were calculated. In Phase 2, conceptual and operational definitions were formulated. In addition, their content was validated with a focus group in Phase 3. In Phase 4, the results were applied in clinical practice and convergence with the National Institute of Health Stroke Scale was verified. The reliability was measured by Cronbach’s alpha. Of the 22 initial indicators, 6 were excluded. The focus group suggested changes in the definitions and the exclusion of two indicators. In Phase 4, only 13 indicators were validated due to the impossibility of measuring intracranial pressure. A strong correlation between the two scales and agreement among all the indicators were observed. Following the specialists’ review, the nursing outcome was reliable and clinically validated with 13 indicators: consciousness, orientation, language, central motor control, cranial sensory and motor function, spinal sensory and motor function, body temperature, blood pressure, heart rate, eye movement pattern, pupil size, pupil reactivity, and breathing pattern.

## 1. Introduction

Stroke is a severe, disabling cerebrovascular disease (CVD) that can lead to serious impairment and death. It requires immediate multi-professional intervention in health services, with rapid diagnosis and individualized, specialized, and quality nursing care. It is necessary to use the nursing process and neurological assessment scales to facilitate nursing care planning for stroke patients [1,2]. Therefore, early nursing care is essential, using reliable and valid instruments to identify neurological changes.

Among them, the Nursing Outcomes Classification (NOC) stands out for identifying nursing outcomes (NO) with indicators and scales capable of evaluating the patient’s condition along the continuum and the effectiveness of the care plan [3,4].

Each NO has a five-point Likert scale to assess the listed indicators. The scales allow measurement at any point on the continuum so that the fifth point reflects the condition of the patient that is most desired, facilitating the identification of changes in their state through different scores throughout time. The use of the NOC makes it possible, in this way, to monitor the improvement, worsening, or stagnation of the patient’s condition [5].

Due to its clear standardized approach and ease of use in conjunction with the NANDA-I (nursing diagnoses) and Nursing Interventions Classification (nursing interventions) taxonomies, the NOC has been considered a facilitator for communication between nurses and clinically useful for practice care [4].

The use of the NO “Neurological Status” (code: 0909) requires further research. This NO has 22 indicators, classified according to a five-point Likert scale. It is defined as the “ability of the peripheral and central nervous systems to receive, process, and respond to internal and external stimuli” [3] (p. 383) and can be used from the acute to chronic stages of cerebrovascular diseases, especially stroke [6,7]. This NO was proposed by the Center for Nursing Classification and Clinical Effectiveness (CNC), a self-supporting center located within the University of Iowa College of Nursing. No previous study was done with the aim of validating this NO.

As well as the various nursing taxonomies, the NOC needs periodic review to adapt its components to the current literature. In this context, validation studies are of great importance to determine the accuracy and effectiveness of nursing classification systems. Some validation studies of NO in patients with cardiovascular diseases have been carried out [8,9,10,11,12]. 

A review study of the knowledge produced from the NOC identified the significant increase in studies on this topic, which highlights the space that taxonomy has been conquering in clinical practice and scientific research. In addition, most studies involving the NOC have focused on the review of concepts and on building operational definitions for the NOC indicators, with a concern to legitimize the elements that make up the classification. New validation studies involving nursing outcomes and their indicators, and using new designs, must be carried out [13].

There are other validated and accurate scales worldwide for assessing stroke patients. The National Institute of Health Stroke Scale (NIHSS) is the best known and most referenced protocol and is used in several countries. The NIHSS measures the neurological severity of the disease and effectively predicts the patient’s prognosis. However, this scale has limitations and weaknesses in its application. For example, there is a need for health professionals to undergo prior training to become able to use the instrument in their practice. Factors such as comorbidities, location, and size of the brain lesion, as well as complications already installed at the time of assessment, can generate a final scale score that does not reflect the patient’s actual clinical status. These factors alter the sensitivity of the NIHSS scale to the patient’s actual clinical status [14,15]. The NOC and the NIHSS have similar evaluation indicators despite the limitations mentioned above. Thus, the NIHSS can be used convergently with the NOC, making nursing outcomes even more accurate.

Given the above, the following hypothesis was raised: the nursing outcome “Neurological Status,” supported by operational definitions formulated and submitted to a validation process with specialists, is adequate to evaluate the neurological status of patients with CVD when compared to the NIHSS, which is considered the gold standard [16]. Therefore, the objective of this study was to validate the nursing outcome “Neurological Status” for patients with cerebrovascular diseases.

It is believed that the present study will be relevant to the clinical practice of nurses, as it allows for the review of NO that can be used in the daily care of stroke patients. For nursing research and science, it may deepen the methods used for validating nursing outcomes, and reviews a specific nursing outcome in the field of neuroscience.

## 2. Materials and Methods

### 2.1. Design 

A methodological study was performed in four phases: (1) evaluation of the indicator’s relevance by specialists; (2) formulation of conceptual and operational definitions; (3) validation of definitions by specialists; and (4) clinical validation by a pilot test.

Methodological studies involve the investigation of methods to obtain and organize data through rigorous research methods, testing of interventions, and sophisticated procedures for obtaining data. They deal with developing, validating, and evaluating research tools and methods [17]. This research followed the recommendations of the COSMIN reporting guideline for studies on measurement properties of patient-reported outcome measures [18]. 

### 2.2. Phase 1—Evaluation of Indicator Relevance by Specialists

Relevance measures to what extent an indicator is consistent and represents the concept in question, which, in the case of the study, is how well the indicator represents the neurological status of patients with cerebrovascular disease.

To evaluate the relevance of the 22 indicators of the NO “Neurological Status,” seven specialists were intentionally selected [18,19], meeting the following inclusion criteria: being an intensive care nurse or physician, and having at least one year of practice in providing care to stroke patients. Four participants were nurses and three were physicians—two clinical neurologists and one intensivist—with at least two years of experience in providing care to stroke patients. The study was conducted with an interdisciplinary approach, since the different members of the healthcare team may use the instrument in future practices and not only with nurses.

The modified kappa coefficient (MKC) was used to evaluate the degree of agreement (reliability and precision) among the specialists regarding the relevance of the indicators. Values over 0.75 indicate excellent agreement, values between 0.75 and 0.40 indicate average agreement, and values below 0.40 indicate poor agreement [20]. The content validity index (CVI) was also used to evaluate the percentage of agreement among specialists. In this study, the clinical indicators with a MKC above 0.75 and a CVI above 80% were considered relevant [21].

### 2.3. Phase 2—Formulation of Conceptual and Operational Definitions

A literature review of anatomy, semiology, and neurology books was carried out to formulate the conceptual and operational definitions of the relevant indicators in Phase 1. As in a previous study with NO in physiology [11], this was necessary because the method to evaluate indicators was not described in detail in scientific articles found in the electronic databases. All the definitions were formulated by the main researcher and refined together with the supervisor. 

### 2.4. Phase 3—Validation of Definitions by Specialists

Following previous research [5,18], the focal group methodology was used to validate the definitions and identify suggestions, including the change of labels/names, grouping or exclusion of elements, and to check the accuracy and clarity of each definition.

The accuracy evaluates to what extent the indicator/definition performs the proposed measurement without mistakes and conceptualizes the domains objectively, without generating other meanings [18,22].

The group was composed of seven specialists selected by convenience [18,21], other than those in Phase 1. The professionals invited met the following criteria: at least one year of experience in providing care to stroke patients and using nursing taxonomies; or having scientific publications in nursing taxonomies or validation studies. The group included a nursing professor, three master’s students in health sciences with a focus on nursing, a doctoral student in health sciences with a focus on scale validation, and two specialist nurses who provide care to patients with cerebrovascular diseases. 

All specialists received the material by e-mail for an initial analysis. Two four-hour face-to-face meetings were held in which the content of the definitions was evaluated, thereby being considered a 100% consensus among the evaluators. 

### 2.5. Phase 4—Clinical Validation by a Pilot Test

#### 2.5.1. Participants

The patients were selected from a single healthcare facility in the countryside of São Paulo, Brazil, and were either hospitalized in an adult in-patient or intensive care unit or in out-patient follow-up treatment. Fifty-nine patients were included in the stipulated period for data collection, meeting the following criteria: diagnosis of ischemic or hemorrhagic stroke, transient ischemic attack (TIA), or cerebral venous thrombosis (CVT); age above 18; and ability to communicate or having a legal representative who could provide information about the health-disease process. 

#### 2.5.2. Data Collection

The data were collected between November 2018 and February 2019 through anamnesis, physical examination, and analysis of medical records by a nurse with five years of experience providing care to stroke patients and using nursing taxonomies. In this phase, the conceptual and operational definitions of the indicators, as elaborated previously, were used.

Validated equipment was used to verify clinical data such as blood pressure and body temperature, which were measured namely with the Onrom HEM-7122^®^ and Onrom MC-720^®^ digital forehead thermometers, respectively, to increase measurement accuracy. The clinical indicator intracranial pressure was not measured in the data collection as no patient had the measuring catheter installed at the time.

#### 2.5.3. Instruments

The sociodemographic and clinical characterization instrument included sociodemographic data (gender, age, ethnicity, marital status, education, and family income) and clinical data (type of cerebrovascular disease, treatment, and evaluation unit). The instrument used to measure the nursing outcome “Neurological Status” contained 14 indicators that were validated in the previous steps, with all formulated definitions.

The National Institutes of Health Stroke Scale was used for convergent validity to test the hypothesis of negative correlations between the NO “Neurological Status” and NIHSS. This scale aims to evaluate the neurological severity of a stroke and predict the prognosis of stroke patients by using 11 items measured by a 3- to 4-point Likert scale, with an overall score varying from 0 to 42, in which higher scores represent a greater severity of neurological status [16,23]. The nurse responsible for the data collection underwent online training with the Medical School of the University of Porto, Portugal [24].

#### 2.5.4. Statistical Analysis

The data were tabulated in Excel for Windows 2010^®^ and analyzed using SAS^®^ software version 9.4 (SAS software Brazil, Sao Paulo, Brazil). A descriptive analysis of the quantitative and qualitative variables was performed, and then data normality was verified using the Shapiro-Wilk test.

Given the asymmetric distribution of the variables, Spearman’s coefficient was used to check the correlation between the NO indicators and the NIHSS score, and the following classification was adopted: 0.10 to 0.29 (weak correlation), 0.30 to 0.49 (moderate correlation), and greater than or equal to 0.50 (strong correlation) [25]. The test could not be performed for all indicators due to the sample size of each one and because the testing assumptions were not met.

Next, the NO indicator scores were grouped (1, 2, and 3—altered indicator/nursing intervention required; 4 and 5—normal indicator/no nursing intervention required), as well as the NIHSS items (1—unchanged item; and 2, 3, and 4—item indicates some change), and then the agreement between them was checked using Cohen’s kappa coefficient. The following classification was used for this: 0 to 0.20: slight agreement; 0.21 to 0.40: fair agreement; 0.41 to 0.60: moderate agreement; 0.61 to 0.80: substantial agreement; and 0.81 to 1.00: almost perfect agreement [26]. The level of significance for all tests was 5%.

The reliability of the NO scale was verified by analyzing the internal consistency. The Cronbach’s α coefficient was calculated for the overall scale and the scale, excluding each item individually. The evidence of satisfactory internal consistency is a value greater than or equal to 0.07. The item-total correlation was measured to complement the assessment, whose values were equal to or above 0.30 as recommended [18,27].

### 2.6. Ethical Considerations

The consent of the research institution was granted by the Research Ethics Committee of the University of Campinas (opinion issued on 18 April 2018, Ref. No. 2.606.228) and complied with the Declaration of Helsinki. The specialists, patients, and caregivers were informed about study objectives and procedures; then, written consent was obtained for inclusion.

## 3. Results

### 3.1. Phase 1

Following the evaluation of the 22 NOC indicators, 6 indicators were excluded due to the CVI and MKC values. There were changes to the title of four indicators, for the specialists argued that even though they are relevant to the assessment, the terminology used was not common in clinical practice (Table 1). 

### 3.2. Phases 2 and 3

The conceptual and operational definitions and their magnitudes were formulated for the 16 indicators resulting from Phase 1. The focus group made it possible to refine the definitions of all indicators. The *breathing rate* indicator was grouped under *breathing pattern*, as the specialists considered the latter more comprehensive for the evaluation. The *radial pulse rate* indicator was excluded because the specialists considered that the *heart rate* indicator would be more accurate given the patients’ clinical condition. The specialists refined the material and a content-validated instrument with 14 indicators for the NO “Neurological Status” was produced in this phase (Table 2 and Appendix A). 

### 3.3. Phase 4

The patients were male (50.9%), had a mean age of 58.6 years (SD = 14.2), white (62.7%), and had a partner (57.6%), complete elementary schooling (30.5%), and average family income of R$ 2119.00 (SD = 103.8). Ischemic stroke patients (77.9%) were the majority, 40.7% of whom were treated with intravenous thrombolysis, while the rest received regular clinical treatment. Of the 13.6% with hemorrhagic stroke, only 3.4% received surgical intervention. Of the remaining subjects, 5.1% had CVT and 3.4% had TIA and continued clinical treatment. Regarding the follow-up treatment locations, 67.8% were in the out-patient clinic, 25.4% in the intensive care unit, and 6.8% in clinical wards.

Thirteen indicators were clinically validated for the NO “Neurological Status” (Table 2) in a pilot test. When analyzing the correlation measured by Spearman’s coefficient between the NO “Neurological Status” indicators and the NIHSS score, a negative correlation was observed at a moderate to strong magnitude, i.e., convergent validity between the two scales. In turn, when examining the individual agreement among the NO indicators, all showed some degree of agreement with the NIHSS items. It is noteworthy that seven NO indicators showed a moderate to substantial level of agreement with eight NIHSS items in the evaluation of the neurological status of stroke patients, namely: *consciousness*, *orientation*, *pupil size*, *pupil reactivity*, *spinal sensory and motor function*, *language,* and *breathing pattern* (Table 3).

Regarding the measure of internal consistency, the final scale had a Cronbach’s alpha equal to 0.79 (Table 4), which shows adequate reliability. As for the item-total correlation, this was less than 0.30 only for the *blood pressure* and *body temperature* indicators, demonstrating a lack of relationship with nursing outcome “Neurological Status”. Thus, it is recommended to exclude these two indicators based on the reliability analysis.

## 4. Discussion

This study presented the validation process of the NO “Neurological Status” that proved viable to evaluate patients with cerebrovascular diseases with different levels of severity. In addition, it has followed phases used in recent studies, such as the formulation of definitions and content and clinical validation using different methods. The last phase examined the convergence of the NO in question with NIHSS, namely the correlation between the scales scores and agreement between their items, as also observed in other studies using different scales to evaluate stroke severity [28,29]. 

A highlight of this study is the possibility of producing practice tools that are specific and sensitive to nursing interventions, easy to apply, provide outcomes that are compatible with the patient’s clinical status [3,4,30], and contribute to an enhanced quality of nursing care [31].

Also, the NO showed convergence validity with the NIHSS, which is considered the gold standard for stroke severity measures. The validation of the NO “Neurological Status” and its comparison with the NIHSS scale can help nurses and other healthcare team members assess patients with stroke. The assessment of the NO mentioned above in clinical practice favors clinical assessment research, policy development, and the interdisciplinary healthcare team.

Following the NOC refinement process, of the 22 initial indicators [3], 14 were considered relevant for neurological evaluation. Besides considering the list of indicators, it is important to reduce subjectivity in practical measurement. However, the lack of criteria to assist this type of evaluation is underlined as a limiting factor to NOC use [4,13]. This deficiency has been overcome by the use of conceptual and operational definitions validated by specialists [8,9,10,11,12].

When examining the indicators individually, it was found that *level of consciousness* helps predict the prognosis and severity of the disease in the first hours. This indicator is associated with patients in a state of agitation or coma in the acute phase of stroke or in cases of CVT, which have critical conditions and a high risk of death. The evaluation of *orientation* is included alongside this indicator [29,32].

The *pupil size* and *pupil reactivity* indicators make it possible to differentiate cerebrovascular diseases since there are specific changes directly linked to brain damage. In the case of ischemic stroke, the pupils tend to present anisocoria; in hemorrhagic strokes, in turn, they tend to develop mydriasis [33]. *Eye movement pattern* is also related to the severity of brain damage and is sensitive to changes in clinical condition as it indicates the integrity of the oculomotor nerve [34]. Therefore, in measuring these indicators, nurses can predict the severity of the disease before the onset of another hemodynamic change.

Whether hemodynamic instability occurs or not, the indicators related to vital signs need to be monitored. *Blood pressure* and *heart rate* allow for adequate maintenance of cerebral perfusion, thereby favoring tissue recovery. In cases of ischemic stroke, it is necessary to maintain the mean blood pressure at higher values—90 to 110 mmHg—while, in hemorrhagic stroke, blood pressure contributes to the prevention and control of bleeding—so it must be maintained at lower values [35]. Accordingly, when nurses can assess the level of cardiovascular/cerebrovascular alteration through a validated and effective scale, they will be able to outline priority nursing interventions to improve specific indicators and achieve a better outcome.

The need for immediate interventions to achieve better outcomes can also be applied to *body temperature*, a potential indicator of impaired clinical evolution in neurological patients. Hyperthermia causes secondary neuronal damage and increases mortality, while hypothermia can be used to reduce brain damage. Therefore, maintaining body temperature at lower levels reduces the brain’s oxygen demand, thereby providing neurological protection against ischemia [36].

The early evaluation of language, central motor control, and spinal sensory and motor function is important to prevent future chronic complications as some indicators of this NO may be more sensitive to interventions during the chronic stage of the disease, given that 80% of stroke patients have some cognitive or motor impairment leading to different and permanent disabilities [9,37]. 

The *intracranial pressure* indicator is crucial in evaluating critically ill patients, especially in cases of risk of cerebral edema and bleeding [38]. Therefore, further studies on the NO “Neurological Status” in the acute phase of the disease are needed.

This study observed that the neurological status of patients with cerebrovascular diseases was less compromised, which can be associated with the fact that the patients were mainly in the chronic phase of the disease.

Despite the strict criteria of each phase, the study presented limitations. First, the convenience sampling and the data collection at a single hospital limit the generalization of our findings. However, the study was a pilot project and further research must be carried out in different world regions. Also, there was difficulty obtaining the consent of inpatients and there was a predominance of patients in out-patient care. The lack of studies on this NO limited the comparison of the findings. 

Scales that quickly measure a patient’s clinical condition with stroke in an emergency situation help nurses trace the desired results and propose effective interventions to improve the patient’s clinical condition. This may be decisive in taking a priority decision to improve the clinical condition and may help to avoid future complications and situations with imminent risk of death.

## 5. Conclusions

Following a review by specialists, the NO “Neurological Status” was clinically validated in a convergent way with the NIHSS for patients with cerebrovascular diseases with an adequate reliability, resulting in 13 indicators, namely: consciousness, orientation, language, central motor control, cranial sensory and motor function, spinal sensory and motor function, body temperature, blood pressure, heart rate, eye movement pattern, pupil size, pupil reactivity, and breathing pattern.

The NIHSS is well defined and easy to use by a wide range of practitioners. By identifying the convergent validity of this scale with the NOC, a new nursing-specific assessment scale is proposed that can also be extended to other health professionals. The NO “Neurological Status” allows the assessment of neurological clinical indicators of patients with cerebrovascular diseases, ensuring a more accurate and reliable evaluation over time. In addition, it allows for determining the effectiveness of applied independent or collaborative nursing interventions. The research contributes to nursing practices by strengthening the use of nursing taxonomies, as a validated NO has been made available. We emphasize the contribution to nursing science in developing new methods for validating the NOC indicators. Further studies need to be carried out to assess the sensitivity of NO in situations where the assessment measured by the NIHSS does not appear accurate.

## Figures and Tables

**Table 1:** Agreement between the experts based on the relevance criteria for indicators of the nursing outcome “Neurological Status”.

Code	Indicators	CVI *	MKC **
	Consciousness	100.00	1.00
90902	Central motor control	100.00	1.00
90903	Cranial sensory and motor function	85.71	0.85
90904	Spinal sensory and motor function	100.00	1.00
90905	Autonomic function	**71.43**	**0.66**
90906	Intracranial pressure	100.00	1.00
90907	Communication appropriate to situation	85.71	0.85
90908	Pupil size	85.71	0.85
90909	Pupil reactivity	85.71	0.85
90910	Eye movement pattern	85.71	0.85
90911	Breathing pattern	85.71	0.85
90913	Sleep-rest pattern	**71.43**	**0.66**
90914	Seizure activity	**57.14**	**0.41**
90915	Headaches	**57.14**	**0.41**
90917	Blood pressure	85.71	0.85
90918	Pulse pressure	**42.86**	**0.21**
90919	Respiratory rate	85.71	0.85
90920	Hyperthermia	85.71	0.85
90921	Apical heart rate	85.71	0.85
90922	Radial pulse rate	85.71	0.85
90923	Cognitive orientation	85.71	0.85
90924	Cognitive status	**42.86**	**0.21**

* Content Validity Index; ** Modified kappa coefficient; Note: Bold indicates the excluded indicators.

**Table 2:** Summary of “Neurological Status” indicators validated at each phase of the study.

Indicators	Phase 1	Phase 2	Phase 3	Phase 4
Consciousness	✔	✔	✔	✔
Central motor control	✔	✔	✔	✔
Cranial sensory and motor function	✔	✔	✔	✔
Spinal sensory and motor function	✔	✔	✔	✔
Autonomic function	Exclude	Exclude	Exclude	Exclude
Intracranial pressure	✔	✔	✔	Unvalued
Communication appropriate to the situation	Change to *Language*	✔	✔	✔
Pupil size	✔	✔	✔	✔
Pupil reactivity	✔	✔	✔	✔
Eye movement pattern	✔	✔	✔	✔
Breathing pattern	✔	✔	✔	✔
Sleep-rest pattern	Exclude	Exclude	Exclude	Exclude
Seizure activity	Exclude	Exclude	Exclude	Exclude
Headaches	Exclude	Exclude	Exclude	Exclude
Blood pressure	✔	✔	✔	✔
Pulse pressure	Exclude	Exclude	Exclude	Exclude
Respiratory rate	✔	Grouped to *Breathing pattern*	Exclude	Exclude
Hyperthermia	Change to *Body temperature*	✔	✔	✔
Apical heart rate	Change to *Heart rate*	✔	✔	✔
Radial pulse rate	✔	Exclude	Exclude	Exclude
Cognitive orientation	Change to *Orientation*	✔	✔	✔
Cognitive status	Exclude	Exclude	Exclude	Exclude

**Table 3:** Values of agreement and correlation between the indicators and the nursing outcome “Neurological Status” with the items and the total score of the National Institute of Health Stroke Scale.

Indicators	Mean	NIHSS Items *													NIHSS **
		1a	1b	1c	2	3	4	5	6	7	8	9	10	11	
Consciousness	4.89	0.38	-	0.19	-	-	-	-	-	-	-	-	-	0.48	-
Orientation	4.55	-	0.63	0.63	-	-	-	-	-	-	-	-	-	-	−0.63
Central motor control	3.73	-	-	0.37	-	-	-	0.58	0.59	0.52	-	-	-	-	−0.68
Cranial sensory and motor function	3.73	-	-	0.16	0.23	0.24	0.29	-	-	-	-	0.31	0.37	0.08	−0.31
Spinal sensory and motor function	4.20	0.19	0.35	0.35	-	-	0.32	0.68	0.61	0.61	0.59	0.39	-	-	−0.72
Language	4.18	-	-	0.41	-	-	-	-	-	-	-	0.31	-	-	−0.67
Pupil size	4.66	0.39	0.34	0.56	0.24	-	-	-	-	-	-	0.38	-	0.19	−0.35
Pupil reactivity	4.81	-	0.12	0.36	0.48	0.24	-	-	-	-	-	0.14	-	-	-
Eye movement pattern	4.91	-	-	0.36	-	-	-	-	-	-	-	-	-	0.37	-
Breathing pattern	4.82	0.65	0.32	0.12	-	-	-	0.11	-	0.14	-	0.36	0.14	-	-
Blood pressure	3.81	-	-	-	-	-	0.13	0.25	0.18	0.19	0.14	-	-	-	ns
Body temperature	4.81	-	-	0.14	-	-	-	-	-	-	-	0.14	0.14	-	-
Heart rate	4.83	-	-	-	0.48	0.24	-	-	-	-	-	-	0.14	-	-
Nursing outcome “Neurological Status”	4.45	-	-	-	-	-	-	-	-	-	-	-	-	-	−0.75

* Cohen’s Kappa Coefficient; ** Spearman’s coefficient; Note: Ns—not significant; Items NIHSS: 1a. Level of Consciousness (LOC); 1b. LOC Questions; 1c. LOC Commands; 2. Best Gaze; 3. Visual; 4. Facial Palsy; 5. Motor Arm; 6. Motor Leg; 7. Limb Ataxia; 8. Sensory; 9. Best Language; 10. Dysarthria; 11. Extinction and Inattention (formerly Neglect).

**Table 4:** Internal consistency (Cronbach’s alpha nursing outcome “Neurological Status” for patients with cerebrovascular diseases.).

Indicators	Corrected Item-Total Correlation	Cronbach’s Alpha If Item Deleted
Consciousness	0.42	0.78
Orientation	0.61	0.76
Central motor control	0.53	0.77
Cranial sensory and motor function	0.49	0.77
Spinal sensory and motor function	0.46	0.78
Language	0.80	0.75
Pupil size	0.49	0 77
Pupil reactivity	0.34	0.79
Eye movement pattern	0.32	0.79
Breathing pattern	0.66	0.76
Blood pressure	0.15	0.80
Body temperature	0.07	0.81
Heart rate	0.30	0.79
Nursing outcome “Neurological Status”		0.79

## Data Availability

The data presented in this study are available on request from the corresponding author.

## Appendix A

**Table A1:** Operational definitions of the indicators of the nursing outcome “Neurological Status” validated in the focus group.

Consciousness—Speak to the patient in a normal tone of voice, gradually increasing it and using tactile and painful stimuli if there is no response. The sites to apply painful stimuli are the sternum, nail bed, and glabella. Magnitudes: 1. Coma; 2. Stupor or torpor; 3. Obnubilation; 4. Lethargy or drowsiness; 5. Alertness or wakefulness
Orientation—Ask the patient questions about personal identification, day of the week, month, year, place of residence, and current location. Magnitudes: NA—Aphasia or inability to communicate; 1. Disorientation in time, space, and person; 2. Disorientation in person and space; 3. Disorientation in time and space; 4. Disorientation in time; 5. Orientation regarding time, space, and person
Central motor control—Evaluate musculoskeletal activities by muscle tone, strength, coordination, gait, superficial cutaneous plantar reflex, posture, and involuntary movements. Magnitudes: 1. Change in 6 or 7 items; 2. Change in 4 or 5 items; 3. Change in 2 or 3 items; 4. Change in 1 item; 5. No change
Cranial sensory and motor function: Evaluate facial sensitivity and movement and visual and hearing acuity. Magnitudes: NA—Patient in a coma or RASS -4 and -5 deep sedation; 1. Change in 4 items; 2. Change in 3 items; 3. Change in 2 items; 4. Change in 1 item; 5. No change
Spinal sensory and motor function: Evaluate the four limbs’ motor function and tactile sensitivity. Magnitudes: NA—Patient in a coma or RASS -4 and -5 deep sedation; 1. Absence of movement in the whole body and/or complete loss of sensitivity; 2. Absence of movement in the whole body and decreased sensitivity; 3. Absence of movement in the hemibody (hemiplegia) or a limb with reduced sensitivity; 4. Movement preserved in the whole body with decreased sensitivity in one limb; 5. Movement of the whole body and preserved sensitivity.
Language: Do the following test: (a) Show the patient a pen and a watch and ask him or her to name both objects, assigning 1 mark for each correct answer; (b) Ask the patient to repeat the phrase “neither here nor there nor anywhere,” assigning 1 mark if correctly repeated; (c) Do the three commands test, asking the patient to “Get the sheet of paper with your right hand, fold it in half, and place it on the table,” assigning 1 mark for each command correctly performed. Magnitudes: NA—Patients in a coma or RASS -4 and -5 deep sedation; 1. Score below 3 in the tests; 2. Score 3 in the tests; 3. Score 4 in the tests; 4. Score 5 in the tests; 5. Score 6 in the tests
Intracranial pressure: Consider the value shown on a monitor identified as ICP, measured through the specific catheter inserted by neurosurgeons between the meninges for this measurement. Magnitudes: NA—Patients with no measurement catheter; 1. ICP above 60 mmHg; 2. ICP between 41 and 60 mmHg; 3. ICP between 21 and 40 mmHg; 4. ICP between 16 and 20 mmHg; 5. ICP between 0 and 15 mmHg
Pupil size: Evaluate by directly examining the pupils, opening the patient’s eyelids, and measuring pupil diameter using a millimeter ruler (pupilometer). Magnitudes: NA—Previous change; 1. Bilateral mydriasis (pupils > 4 mm); 2. Unilateral mydriasis (pupil > 4 mm); 3. Bilateral myosis (pupils < 2 mm); 4. Unilateral myosis (pupil < 2 mm); 5. Normal-sized and isochoric pupils (equal)
Pupil reactivity—Evaluate direct and indirect photomotor reflexes of both eyes. Magnitudes: NA—Previous change; 1. Absent direct and indirect photomotor reflex bilaterally; 2. Present direct photomotor reflex unilaterally and absent indirect photomotor reflex bilaterally; 3. Present direct photomotor reflex bilaterally and absent indirect photomotor reflex bilaterally; 4. Present direct photomotor reflex bilaterally and absent indirect photomotor reflex unilateral; 5. Present direct and indirect photomotor reflex bilaterally.
Eye movement pattern: Evaluate using the following tests and criteria: (a) Ask the patient to follow your index finger with the eyes, without moving the head, to the left, right, down, and up. Observe the movements and possible gaze deviation; if there is gaze deviation or paralysis, assign a score of 2; (b) Do the eye convergence test with the patient looking forward and with the head still, gradually bring your index finger close to the patient’s eyes; if there is no gaze convergence, assign a score of 1; (c) Complaint of diplopia or presence of involuntary eye movements during the tests or with the patient at rest, assign a score of 1. Magnitudes: NA—Patients in a coma or RASS -4 and -5 deep sedation; 1. Score > 3; 2. Score 3; 3. Score 2; 4. Score 1; 5. No change
Breathing pattern: Evaluate breathing amplitude, breathing rate, chest expansion, and breathing rhythm. Magnitudes: NA—Mechanical ventilation in controlled modes; 1. Change in 4 items; 2. Change in 3 items; 3. Change in 2 items; 4. Change in 1 item; 5. No change.
Blood pressure: Check the blood pressure value by the invasive method (gold standard) if a catheter is inserted directly into the artery or by the non-invasive method using an automated blood pressure monitor using the oscillometric technique Magnitudes: 1. SBP ≥ 180 or SBP ≤ 80/DBP ≥ 110 or DBP ≤ 40; 2. 160 ≤ SBP ≤ 179 or 81 ≤ SBP ≤ 85/100 ≤ DBP ≤ 109 or 41 ≤ DBP ≤ 45. 3. 140 ≤ SBP ≤ 159 or 86 ≤ SBP ≤ 90/90 ≤ DBP ≤ 99 or 46 ≤ DBP ≤ 50. 4. 121 ≤ SBP ≤ 139 or 91 ≤ SBP ≤ 100/81 ≤ DBP ≤ 89 or 51 ≤ DBP ≤ 60. 5. 101 ≤ SBP ≤ 120/61 ≤ DBP ≤ 80
Body temperature: Bring the forehead thermometer close to the front area of the patient’s head, at a distance of 1 to 3 cm, and wait for the body temperature measurement to appear on the screen. Magnitudes: 1. T > 39 °C or T < 34.4 °C; 2. 38.6 < T < 39 °C or 33.9 < T < 34.4 °C; 3. 37.5 < T < 38.5 °C or 34.5 < T < 34.9 °C; 4. 37.1 < T < 37.4 °C or 35 < T < 35.4 °C; 5. 35.5 < T < 37 °C
Heart rate: Auscultate and count the beats per minute (bpm) at the apex of the heart. Magnitudes: NA—Patients in a coma or RASS -4 and -5 deep sedation; 1. HR> 160 bpm OR HR < 40 bpm; 2. 151 ≤ HR ≤ 160 bpm OR 40 ≤ HR ≤ 44 bpm; 3. 131 ≤ HR ≤ 150 bpm OR 45 ≤ HR ≤ 54 bpm; 4. 101 ≤ HR ≤ 130 bpm OR 55 ≤ HR ≤ 59 bpm; 5. 60 ≤ HR ≤ 100 bpm
